# Testosterone associates differently with body mass index and age in serum and cerebrospinal fluid in men

**DOI:** 10.1111/joim.13509

**Published:** 2022-05-31

**Authors:** Henrik Ryberg, Per Johansson, Anders Wallin, Johan F. Emilsson, Elias Eriksson, Johan Svensson, Claes Ohlsson

**Affiliations:** ^1^ Sahlgrenska Osteoporosis Centre Centre for Bone and Arthritis Research Department of Internal Medicine and Clinical Nutrition Institute of Medicine, Sahlgrenska Academy University of Gothenburg Gothenburg Sweden; ^2^ Department of Psychiatry and Neurochemistry Institute of Neuroscience and Physiology Sahlgrenska Academy University of Gothenburg Mölndal Sweden; ^3^ Department of Pharmacology Institute of Neuroscience and Physiology Sahlgrenska Academy University of Gothenburg Gothenburg Sweden

**Keywords:** endocrinology, steroids, androgens, central nervous system, mass spectrometry

Dear Editor,

Testosterone treatment of hypogonadal men has increased substantially during the last 20 years. Although the indications for treatment of hypogonadism differ between countries, both specific symptoms and serum testosterone below a certain value are in general recommended to be used to diagnose hypogonadal men [1, 2].

The main determinants of serum testosterone have been investigated extensively. There is a strong inverse association between body mass index (BMI) and serum testosterone, while there is only a modest decline of circulating testosterone by age in men [1, 3]. Due to the lack of validated assays for measurements of testosterone in cerebrospinal fluid (CSF), the main determinants of CSF testosterone in men—reflecting the central nervous system (CNS) exposure—are less known. The aim of the present study was to identify determinants of testosterone in CSF of healthy men using a highly sensitive gas chromatography‐tandem mass spectrometry (GC‐MS/MS) method validated for CSF measurements.

## Materials and methods

Blood sampling and lumbar puncture were performed simultaneously in 61 healthy men from Västra Götaland County in Sweden. The collected serum and CSF were stored in –80°C pending analyses. Institutional ethical review board approvals had been obtained, and written consent had been obtained from all patients. The GC‐MS/MS method used for the analyses of serum testosterone has been described previously [4]. For this study, we adapted and validated this method for analyses of testosterone in CSF using 450 µl CSF. The sensitivity (limit of quantification 4.9 pg/ml), accuracy (106% at 7.5 pg/ml), and precision (intra‐assay coefficient of variation of 4.5% at 4.9 pg/ml) were excellent for the testosterone measurements in CSF (Supporting Information Methods). Values are given as the mean ± standard deviation. Correlations were examined using Pearson's correlation coefficient. A two‐sided *p* value < 0.05 was considered significant.

## Results

**Fig. 1:**
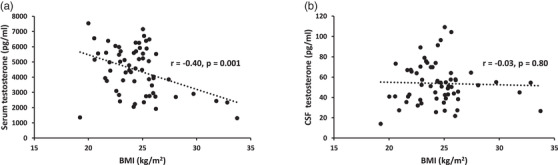
Body mass index (BMI) is inversely correlated with serum but not cerebrospinal fluid (CSF) testosterone. Serum and CSF testosterone were measured in 61 healthy men. Correlations were examined using Pearson´s correlation coefficient (r). A two‐sided p value < 0.05 was considered significant.

The testosterone levels in CSF were on average 1.29 ± 0.45% of the levels in serum (CSF, 53.7 ± 20.0 pg/ml; serum, 4446 ± 1558 pg/ml) in the study population of healthy male volunteers (age, 62.8 ± 18.8 years; BMI, 24.5 ± 2.7 kg/m^2^). We observed a positive correlation between serum and CSF testosterone (*r* = 0.50, *p* < 0.001). As shown in Fig. 1 and Fig. S1, BMI correlated inversely with serum testosterone (*r* = –0.40, *p* = 0.001), while age was not significantly correlated with serum testosterone (*r* = –0.11, *p* = 0.41). In contrast, age correlated inversely with CSF testosterone (*r* = –0.42, *p* = 0.001), while BMI was not associated with CSF testosterone (*r* = –0.03, *p* = 0.80). Associations between CSF testosterone or serum testosterone and weight, height, age, BMI, and body surface area are shown in Table S2.

## Discussion and conclusion

Our validated high sensitivity GC‐MS/MS analyses revealed a direct association between testosterone levels in CSF and blood, indicating that at least part of CSF testosterone is serum derived. This observation contrasts to a recent study—using an immune assay‐based technique for the analyses of CSF testosterone—that did not identify any association between testosterone in serum and CSF in healthy men [5].

The most important finding in the present study is that the determinants of CSF testosterone are different from those of serum testosterone. Although both observationally and causally BMI is strongly inversely associated with serum testosterone, no association between BMI and CSF testosterone was seen in the present study [3, 6]. This indicates that a high BMI does not result in reduced levels of testosterone in the CNS. Instead, testosterone was substantially more reduced by age in CSF compared with in serum. These findings suggest that the regulation of testosterone in CSF differs from that in the circulation, and that an age‐dependent lowering of CSF testosterone—which is not necessarily detectable in serum—might be important for an age‐dependent decline in brain functions in men. Though revealing a positive association between serum and CSF testosterone, our data thus suggest that assessments of circulating testosterone are of only limited relevance for explorations of the brain's exposure to this hormone.

## Conflicts of interest

The authors declare no conflicts of interest.

## Supporting information


**Supplemental Figure 1**. Age is inversely correlated with cerebrospinal fluid (CSF) but not serum testosterone.

**Supplemental Table 1**. Accuracy of testosterone in Human CSF.

**Supplemental Table 2**. Correlations between testosterone and different determinants.
