# Supplementary material for: Testosterone associates differently with body mass index and age in serum and cerebrospinal fluid in men
Source: J Intern Med. 2022 May 31;292(4):684–6. doi: 10.1111/joim.13509 (PMC9543244; doi:10.1111/joim.13509)
Supplement: Supplementary file 1 — Supplemental Figure 1. Age is inversely correlated with cerebrospinal fluid (CSF) but not serum testosterone. [file JOIM-292-684-s002.pdf]

Supplemental Figure 1. Age is inversely correlated with cerebrospinal fluid (CSF) but not serum testosterone.

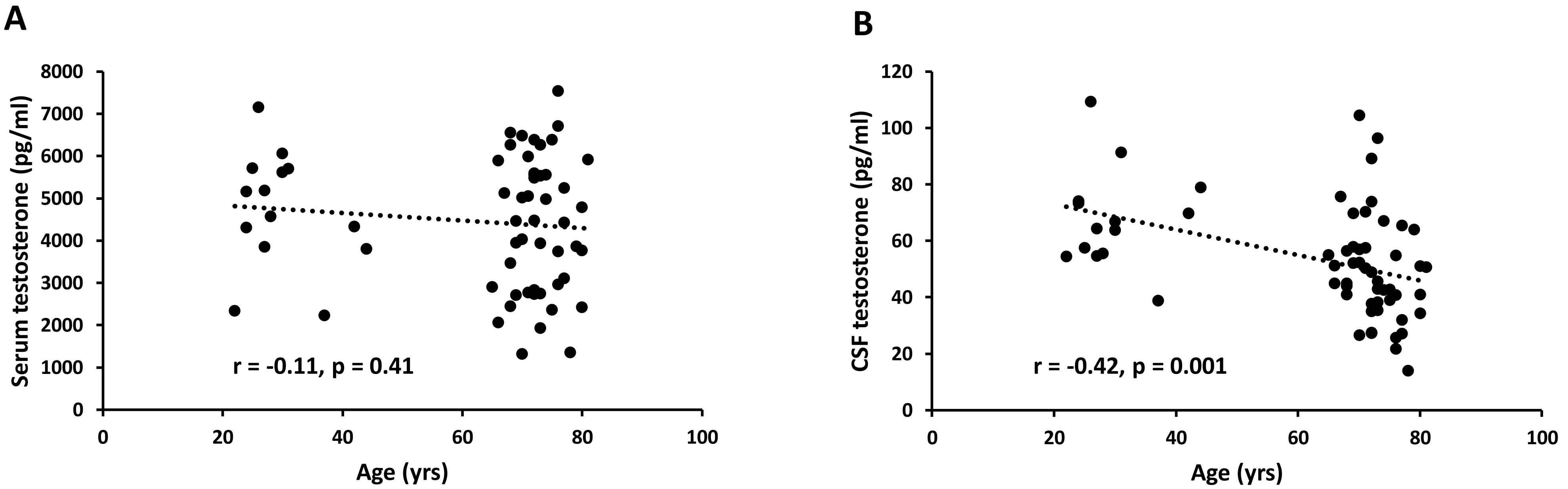

Supplemental Figure 1. Age is inversely correlated with cerebrospinal fluid (CSF) but not serum testosterone. Serum and CSF testosterone were measured in 61 healthy men. Correlations were examined using Pearson´s correlation coefficient (r). A 2-sided P value less than 0.05 was considered significant.
