# Supplementary material for: Testosterone associates differently with body mass index and age in serum and cerebrospinal fluid in men
Source: J Intern Med. 2022 May 31;292(4):684–6. doi: 10.1111/joim.13509 (PMC9543244; doi:10.1111/joim.13509)
Supplement: Supplementary file 2 — Supplemental Table 1. Accuracy of testosterone in Human CSF. [file JOIM-292-684-s003.docx]

**Supplemental Table 2. Correlations between testosterone and different determinants.**

|  | ***Height*** | ***Weight*** | ***BSA*** | ***BMI*** | ***Age*** |
| --- | --- | --- | --- | --- | --- |
| ***Serum T*** | 0.09, p=0.50 | -0.29, p=0.02 | -0.17, p=0.18 | -0.40, p=0.001 | -0.11, p=0.41 |
| ***CSF T*** | 0.14, p=0.27 | 0.05, p=0.72 | 0.10, p=0.45 | -0.03, p=0.80 | -0.42, p=0.001 |

**Supplemental Table 2.** Correlations between testosterone in serum (Serum T) or CSF (CSF T), and different determinants. Correlations were examined using Pearson´s correlation coefficient. BSA stands for the body surface area and was calculated using the formula according to Du Bois and Du Bois [1]; BSA=0.007184×W^0.425^×H^0.725^

**References**

1. Du Bois D, Du Bois EF. A formula to estimate the approximate surface area if height and weight be known. 1916. *Nutrition* 1989; **5:** 303-11; discussion 12-3.
