# Supplementary material for: Testosterone associates differently with body mass index and age in serum and cerebrospinal fluid in men
Source: J Intern Med. 2022 May 31;292(4):684–6. doi: 10.1111/joim.13509 (PMC9543244; doi:10.1111/joim.13509)
Supplement: Supplementary file 3 — Supplemental Table 2. Correlations between testosterone and different determinants. [file JOIM-292-684-s001.docx]

**Supplemental online method for testosterone measurements in CSF**

**Reagents for the GC-MS/MS analyses of testosterone in CSF**

The detailed information on the assay for serum testosterone has been published previously [1]. All reagents used were of pro-analysis grade or better. Pentane, ethyl acetate and heptane were from Merck, Darmstadt, Germany. Testosterone was purchased from Fluka (Buchs, Germany). Isotope-labeled testosterone (testosterone-2,3,4-^13^C_3_) was used as internal standard.

**Calibrators**

The calibrator stock solution was prepared by weighing out and dissolving testosterone in ethyl acetate. Working solutions were prepered in methanol using the stock solution. On each day of analysis, a seven-point calibration curve—including zero—was prepared from the calibrator standard working solutions by dilution in water (1:10). Calibration was performed by determining the peak area ratio between the target analyte and the isotope-labeled internal standard.

**Sample preparation and GC-MS/MS analysis**

For the measurements, 450 µL CSF was used, with 50 µl internal standard and 500 µL of 0.5 M ammonium acetate added to the samples. Testosterone was then extracted using 3 mL 1-chlorobutane and purified on Silica SPE columns (Hypersep Si 500mg, Thermo Scientific, Bellefonte, PA). After washing with ethyl acetate:pentane:heptane (10:45:45, v:v), testosterone was eluted using ethyl acetate:pentane:heptane (50:25:25, v:v), and the organic solvent was evaporated. Oximation was performed using triethylamine in pyridine and pentafluorobenzylhydroxylamine hydrochloride in triethylamine/pyridine at 60°C. After evaporation, esterfication was performed using pentafluorobenzoyl chloride in triethylamine/toluene. The remaining reagents were extracted from the samples using water; the samples were then dried down and reconstituted in 100 µL isooctane prior analysis. The equipment consituted of: an Agilent 7890A GC, an Agilent 7693 autosampler, two 50% phenyl-methyl polysiloxane (DB-17HT) capillary columns (15m×0.25mm internal diameter, 0.15 µm film thickness) and an Agilent 7000 triple quadrupole mass spectrometer (Agilent, Santa Clara, CA). Testosterone was detected with electron capture negative chemical ionization in multiple reaction monitoring (MRM) mode. Ammonia was used as reagent gas. The following transition was used for the quantification: testosterone, 677.2→496.2; testosterone-^13^C_3_, 680.2→499.2; All peaks were automatically integrated using the MassHunter quantitative analysis workstation software from Agilent. Calibration samples with accuracy between 80 and 120% were used for the calibration curves. The calibration curve for testosterone was linear (R^2^ > 0.99).

**Assay performance for CSF testosterone measurements**

Human CSF samples with very low levels of endogenous steroids were merged into two pools with approximatly 10 mL in each, and the endogenous levels were determined. From these pools, samples for sensitivity, precision, and accuracy experiments were created through spiking with stock solutions of testosterone.

Analyses of intraassay coefficients of variation (CVs) were performed using five samples from two pools (with low and high levels of testosterone), demonstrating that the CVs were 4.5% and 0.2% at 4.9 pg/mL and 530 pg/mL, respectively. Analyses of interassay CVs were performed using two pools analysed at eight different occasions, demonstrating that the CVs were 1.4% and 3.5% at 384 pg/mL and 2044 pg/mL, respectively.

Seven different levels in the lower concentration range of testosterone in CSF were evaluated to determine the lower limit of quantification (LLOQ). The LLOQ for testosterone in CSF was 4.9 pg/mL and defined as the lowest peak having a signal at >3 times the noise level, with a CV of less than 20% and an accuracy of 80% -120%.

Aliquots of CSF were spiked with two levels of testosterone to assess accuracy, which was calculated as follows: [(observed value - baseline value)/amount spiked]×100. The accuracies were 106% and 101% in samples spiked with 7.5 pg/mL and 500 pg/ml, respectively (Supplemental Table).

|  | ***Baseline*** *(pg/mL)* | **Spiked** (pg/mL) | ***Observed value*** *(pg/mL)* | ***Accuracy*** *(%)* |
| --- | --- | --- | --- | --- |
| *Low Level* | 1.5 | 7.5 | 9.6 | 106 |
| *High Level* | 26.5 | 500 | 530 | 101 |

**Supplemental Table 1.** Accuracy of testosterone in Human CSF. Accuracy was calculated as follows: [(observed value - baseline value)/amount spiked]×100

**References**

1. Nilsson ME, Vandenput L, Tivesten A*, et al.* Measurement of a Comprehensive Sex Steroid Profile in Rodent Serum by High-Sensitive Gas Chromatography-Tandem Mass Spectrometry. *Endocrinology* 2015; **156:** 2492-502.
